# Arterial hypertension management with conversion enzyme inhibitors in hemodialysis patients

**Published:** 2010-02-25

**Authors:** AC Costea, DO Costea, C David, CN Grasa

**Affiliations:** * Nephrology Clinic, ‘St. John’ Hospital, BucharestRomania; ** Surgery Ⅱ Clinic, Constanta District Emergency HospitalRomania

**Keywords:** arterial hypertension, conversion enzyme inhibitors, chronic renal failure, dialysis

## Abstract

By establishing the renal suppletion procedure, the patients with chronic renal failure also have an acceleration of arterial hypertension phenomena. The management of this situation calls for the understanding of the ethological mechanisms (hypervolemia by the reduction of Na excretion and high rennin secretion) and the adaptation of the therapeutic approach to every patient. An individualized dialysis prescription is imposed, taking into account the residual renal function and an anti–hypertension treatment, in which the role of the conversion enzyme inhibitors (ACE inhibitor) is intensely debated.

In the majority of cases, arterial hypertension (HT) becomes therapeutically controllable with the implementation of renal substitution treatment, the rest of them being candidates for double nephrectomy. The link between the debut of the arterial hypertension and chronic hypertensive nephropathies has been noticed since 1836 along with the research made by Richard Bright. These demonstrated the appearance of a rise in pressure values simultaneously with the evolution of the renal illness. Severe HT, essential HT or other non–renal causes can functionally affect the otherwise healthy kidneys, by inducing nephroangiosclerosis [1, 2]. HT represents an important factor of morbidity and mortality in dialysis patients, especially through the acceleration of the arteriosclerosis' evolution and the evolution with complications, especially those of a cardiovascular cause: cardiac insufficiency, strokes, dissecting aneurysm. 

HT Causes:

1. Chronic glomerulonephritis
2. Systemic lupus erythematosus
3. Systemic atherosclerosis with nephroangiosclerosis
4. Polyarteritis nodossa
5. Diabetic nephropathy
6. Polycystosis
7. Amyloidosis

In the majority of cases, dialysis patients develop terminal conditions of cardiovascular origin, associated with a history of arterial hypertension and advanced sclerosis. Recent studies have shown that there is no direct causality between mean arterial pressure and the survival rate; the left ventricular hypertrophy (LVH) incidence is increased in dialysis patients, and has a significant prognostic value–an important parameter for the monitoring, evaluation and evolution of the disease. 

Even if the pathogenesis of the LVH is of high variety (aortic pressure wave, hormonal imbalance rennin and PTH, renal anemia), it has been shown that this can be limited by administering the correct treatment–the association of dialysis with personal drug therapy, that keeps the arterial pressure values within acceptable limits [3]. It is also of maximum importance that the BP value determination is made, in order to define the BP profile of the dialysis patient. The best time for this evaluation has proven to be a few hours post dialysis, considering the fact that, during the dialysis session, variations generated by the hemodynamic function requirements have been noted, with the volemic status taken into consideration in the pre–dialysis treatment.

Nighttime measurements have shown significant decreases of values because of an autonomically acquired deficiency with a late debut of the chronic renal insufficiency.

There have been documented cases in dialysis, in which, although before the onset of the substitute treatment, the subjects did not present with HT, they developed high values afterwards. These come secondary to their failure to meet their imposed dietary routine, even from technical mistakes during the dialysis procedure like hyperosmolarity of the dialysis patient, inadequate trans–membrane pressure, or intravenous fluids during the dialysis.

Several groups concerning the dialysis patients have been described based on the pathogenesis of the condition that can cross–link in some situations [1]:

## Hydro–saline imbalance HT

Hypervolemia HT seems to be the most frequent cause of hypertension affecting these patients, and this happened due to the complete failure of renal functions; these debuts as a consequence of reestablishing the electrolyte balance, with the secondary expansion of the extracellular liquid volume and the rise of non–exchangeable Na.Table 1

**Table 1:** Consequences of the excess of Na

The rise in the extra cellular liquid volume
The rise in vascular reactivity on the action of pressure hormones
The rise in peripheral vascular system resistance
The rise in oabain–like substance secretion that limit the activity of the Na–K ATPasi
The rise in adrenergic activity
Structural changes of the vascular walls

Therefore, along with the rise in water and Na intake, we observe a rise of the total plasma volume, cardiac output and peripheral vascular resistance, due to the local auto–regulation. These patients have shown low levels of rennin and angiotensin Ⅱ (ATII) values, compared with their volemic status.

The optimum treatment for these forms is established as:

1. Na intake restriction and the close supervision of liquid intake, correlated with the residual dieresis and non–detectable losses–an average of 10ml/kg/day.
2. Lowering plasma Na concentration to 133–135 mEq/L
3. Daily or three times a week dialysis until patient reaches ideal weight

The attention in establishing the patient's ideal weight and the adaptation of dialysis recommendations may assure the correction of pressure values and their stability

## Rennin dependent HT (normal volemia HT)

This type of HT is an altogether different entity, which imposes differential diagnostic issues with the hypervolemia type, making it hard to be diagnosed independently because of the etiological cross–linkage: plasma rennin, ATII, aldosterone, and peripheral resistance are high, with a normal or low serum Na concentration and plasma volume.

Clinically, this form of HT is a malignant development, hard to control, but often with a spectacular result after the administration of ACE inhibitors. For patients on dialysis with therapy–resistant HT, we could incriminate a hyperactivity of plasma rennin even after ultra–filtration and idea–weight adapted dialysis. The administration of ACE inhibitor therapy for these patients has lead to a net improvement of their HT status [5], nevertheless, there have been cases which have required the removal of the rennin source and the reestablishing of pressure balance by– nephrectomy.

Physiopathology studies have recorded paradoxical vascular reactions to any exceeding volemic homeostasis values. Furthermore, the therapeutic loss of salt by dialysis may be the source of ulterior activity of renal vasopressor mechanisms and the generating of HT phenomena hard to control.

The balance between cardiac output and peripheral vascular resistance determines the arterial pressure. Most studies indicate that the cardiac output is normal or above, while extracellular liquid volume is relatively high in patients with chronic renal insufficiency (CRI); systemic vascular resistance is abnormally high relatively to cardiac output, this revealing a switch in the vascular control mechanism towards vasoconstriction. Therefore, patients with CRI show an increased adrenergic tonus and some highly stimulated rennin–angiotensin, endothelin and vasoactive prostaglandins systems. Another aspect observed in some situations is that of diminished nitric oxide (NO) production which reduces the vasodilatation potency.

The link between renal perfusion pressure and salt excretion, which defines pressure natriuresis, has been an element of interest for many studies. In essential HT, a higher renal perfusion pressure is imposed in order to stabilize the sodium balance, but not the same thing happens in patients with renal parenchyma disease, where natriuresis is perturbed by the loss of renal tissue mass. The same effects are also noticed when dealing with endocrine dysfunctions which have an effect on salt excretion (aldosterone–stimulated kidneys), or in the case of diminished renal blood flow and stenosis of the renal artery (Goldblatt kidneys) [9].

The necessity to control arterial blood pressure in patients with terminal renal insufficiency and dialysis patients has imposed the detailed study of the sodium plasma levels in concordance with ultra–filtration. Gathered data include body weight, plasma rennin activity and arterial blood pressure (before and after the administration of ACE inhibitors), on a period of 11 days of treatment. Rigorous ultra–filtration has been performed on these patients set on a low Na diet, in order to obtain a negative Na balance. 

We have observed the increase in plasma rennin concentration, concomitant with sodium extraction, and the values for arterial pressure depends on rennin–ATII system, as we can see after the administration of the ACE inhibitor (captoprilum). In conclusion, the reduction of extracellular liquid volume and sodium plasma levels is essential for the establishing of an efficient control of arterial BP values in patients with CRI.

The relevance of the interaction between sodium and rennin–angiotensin balance in malignant HT is revealed by a study made on a group of subjects with renal dysfunction and accelerated HT. Lowering the BP values after the administration of ACE inhibitors is abruptly altered after the infusion of saline solution, with the establishing of a positive Na balance and the reduced plasma rennin concentration. Administering a strong diuretic induces net sodium excretion, restimulates the rennin synthesis and reestablishes the sensibility to ACE inhibitor therapy. These observations establish the reciprocal relationship between the Na status and the level of circulating rennin.

The latest studies made on dialysis patients who have over 8 years of treatment, have shown that there is no correlation between plasma rennin levels and diastolic BP. In the same time, these studies indicate that severe HT in dialysis patients is an account not only of the nigh plasma rennin activity and ATII generation and extraction, but also of the overlapping excess of salt and water [6, 8]. Therefore, the rennin mechanism interferes with the hydro–electrolytic balance, summing their effects and generating a complicated HT syndrome. These patients associate secondary distance phenomena like vision impairment, retinal hemorrhage, state of conscience dysfunctions or angina and may, at the same time, present anorexia, nausea and intense thirst.

Adapting the therapy by associating an adequate ultra–filtration and the administration of an anti–hypertensive medication based on ACE inhibitors, with a prolonged half time, associated or not with other categories of anti–hypertensive drugs, is the key in managing this type of HT.

ACE inhibitors act directly by interfering with the rennin–angiotensin– aldosterone system, which makes their use become attractive in the therapy of dialysis patients. They are a class of anti–hypertensive drugs generally well tolerated, with low side effects like the lethargy–somnolence syndrome or other effects shown by anti–adrenergic and beta–blocker drugs.

In dialysis patients, the level of prekalicreine is low and that if ACE is high; high plasma rennin activity may be correlated with the increase of aortic pressure. ATII accelerates LVH and that is why the use of ACE inhibitors may stop and even make LVH regress. ACE inhibitors are useful in congestive cardiac insufficiency by reducing the thirst sensation, which is characteristic for high plasma rennin levels, this limiting the weight–gain between dialysis procedures.

The utilization of captoprilum, has been associated with side–effects like:

1. Skin rash
2. Cough
3. Leucopenia or neutropenia
4. Acceleration of anemia

This last effect has become notable after the association of ACE inhibitor therapy with the diminishing of plasma erythropoietin (EPO) or with an increased degree of hemolysis. The explanation for this phenomenon may lie in the direct interference of ATII, with the transcription signal for EPO at a cellular level.

ACE inhibitors reduce arterial blood pressure by lowering peripheral vascular resistance because of the blocked formation of ATII, but there are also other collateral mechanisms. That is why most cases require the administration of more than one class of anti–hypertensive drugs [6].

In conclusion, the management of HT in dialysis patients is linked especially to the disease that causes chronic nephropathy, the substrate of the terminal renal insufficiency, the presence of co–orbidities, the evolution of HT before the start of renal substitution therapy and during dialysis therapy

The optimal attitude is that of continuous evaluation and monitoring of the patient, with the adaptation of therapy by accurately combining the recommendation for dialysis and anti–hypertensive medication, all this in order to reach the aimed arterial blood pressure values.
